# Human Endometrial Stromal/Stem Cells Inhibit Apoptosis in Cisplatin-Induced Acute Kidney Injury in Male Wistar Rats

**DOI:** 10.22074/cellj.2021.7322

**Published:** 2021-10-30

**Authors:** Hadis Zeinali, Mahnaz Azarnia, Peyman Keyhanvar, Reza Moghadasali, Somayeh Ebrahimi-Barough

**Affiliations:** 1. Faculty of Biological Sciences, Kharazmi University, Tehran, Iran; 2. Stem Cell Research Center, Tabriz University of Medical Sciences, Tabriz, Iran; 3. Department of Medical Nanotechnology, School of Advanced Medical Sciences, Tabriz University of Medical Sciences, Tabriz, Iran; 4. Department of Stem Cells and Developmental Biology, Cell Sciences Research Center, Royan Institute for Stem Cell Biology and Technology, ACECR, Tehran, Iran; 5. Department of Tissue Engineering and Applied Cell Sciences, School of Advanced Technologies in Medicine, Tehran University of Medical Sciences, Tehran, Iran

**Keywords:** Acute Kidney Injury, Apoptosis, Cisplatin, Human Endometrial Stromal/Stem Cell

## Abstract

**Objective:**

Acute kidney injury (AKI) is referred to as sudden decline in the function of kidney. Human endometrial stromal/stem cells (hEnSCs) are mesenchymal stem cell (MSC)-like cells, which are suitable candidates for regenerative medicine purposes, yet the effect of hEnSCs on cisplatin-induced AKI has not been studied; therefore, the present study was conducted to investigate this gap in the literature.

**Materials and Methods:**

In this experimental study, hEnSCs were obtained from endometrial biopsy using collagenase I and were then cultured in DMEM/F12 medium. A total of 48 male Wistar rats (150-200 g) were classified into four groups: intact -receiving no treatment, model -receiving 5 mg/kg of body weight cisplatin, as well as phosphate-buffered saline (PBS) and cell -receiving either PBS or hEnSCs for three hours after cisplatin injection, respectively. Biochemical parameters, pathologic scores, apoptosis assay, *Bcl-2* and *Tnf-α* expression were evaluated on day 5.

**Results:**

On day 5 post-transplantation we observed that HEnSCs injection has led to a decrease in both blood urea nitrogen (BUN) and serum creatinine (SCr), compared to the model and PBS groups (0.82 ± 0.03 vs. 1.42 ± 0.06, 1.09 ± 0.05 mg/dl and 61.53 ± 3.07 vs. 116.60 ± 2.12, 112.00 ± 1.35 mg/dl, respectively). The highest levels of pathologic scores were observed in model and PBS groups, while hEnSCs transplantation resulted in a decrease in pathologic scores (149.10 ± 7.03, 141.50 ± 4.68 vs. 118 ± 2.16). HEnSCs significantly decreased the percentage of TUNEL- positive cells in the cell group compared with model and PBS groups (20.37 ±. 3.37 vs. 33.67 ± 1.79, 31.53 ± 1.05 in glomeruli and 15.10 ± 1.47 vs. 42.33 ± 1.72, 39.23 ± 1.61 in tubules). In addition, HEnSCs resulted in upregulation of *Bcl-2* and downregulation of *Tnf-α* in the cisplatin-induced AKI.

**Conclusion:**

Our results showed that injection of hEnSCs may improve AKI through lowering the amount of apoptosis in renal cells.

## Introduction

Acute kidney injury (AKI) is defined as sudden decline in the function of kidneys for waste removal, which is due to renal tubular damage (1). Cell death, as well as functional and morphological changes, occur in sublethal damage caused by AKI (2). About 20% and 33% of hospitalized adults and children, respectively, suffer from AKI, and the mortality rate due to AKI in patients of surgical and medical intensive care units (ICU) is high. Consequently, AKI has attracted much attention among nephrologist and intensive care unit care providers (3, 4). Common treatments used in AKI include pharmacological therapy, dialysis, and renal replacement therapy, but each of these treatments still has certain limitations (5). Dialysis and replacement therapies have no significant effect on the high rate mortality caused by AKI. Dialysis is commonly associated with socioeconomic problems. With regards to replacement therapy, the lack of enough donors and the use of immunosuppressive drugs following kidney transplantation may cause issues for the recipients. Pharmacological therapy has been unsuccessful in terms of recovering lost cells. Therefore, new therapeutic strategies for AKI seem to be instantly required (6).

Among the common experimental models of AKI are toxic models, which are caused by nephrotoxins, such as cisplatin. Cisplatin-induced extensive injury is usually assessed on days 3-5 post-treatment, whereas earlier time points may lead to sub-lethal changes (2). Cisplatin or cis-diamminedichloroplatinum (II) is a chemotherapeutic drug, which contains platinum, carboplatin or oxaliplatin. Platinum in cisplatin compound binds to DNA and results in crosslinking that triggers apoptosis (7). Sodium wasting, disturbance of magnesium reabsorption and water absorption are among other consequences of cisplatin therapy. Additionally, prevention of kidney injury is very important in cisplatin therapy (8, 9). Cisplatin-induced tubular dysfunction is due to apoptosis and necrosis. Tubular cell death, resulting from cisplatin, occurs via multiple signaling pathways and mechanisms that have been considered as potential targets for various clinical treatments (10-14). Two signaling pathways of apoptosis include intrinsic (mitochondrial) and extrinsic (death receptor) pathways. Apoptosis is initiated by the extrinsic signaling pathway, which begins with a cell death signal or death ligand docking on tumor necrosis factor (TNF) superfamily death receptors (15). The intrinsic pathway of apoptosis, on the other hand, is regulated by B-cell lymphoma-2 (Bcl-2) protein family, as the Bcl-2 family members play different roles in intrinsic death pathway. For instance, anti-apoptotic Bcl-2 proteins inhibit pro-apoptotic members of the family (BAX and BAK) and thereby inhibit apoptosis (15, 16).

Multiple studies have demonstrated enormous potentials for mesenchymal stem cells (MSCs) to repair the damaged tissues via infusion and paracrine signaling (17-19). Human endometrial stromal/stem cells (HEnSCs) are MSC-like cells obtained from the endometrium. HEnSCs can be isolated easily, expand rapidly, have no major technical and ethical issues, and have a high clonogenicity; and therefore, are suitable candidates for therapeutic and tissue engineering purposes (20, 21). The repair of soft tissue defects is a promising regenerative capacity of the hEnSCs (22). Transplanted EnSCs in the peri-infarct area increase cellular proliferation, but decrease apoptosis via AKT, ERK1/2, STAT3 activation and p38 inhibition (23). Pabla and Dong have found that cisplatin exerts its ultimate destructive effects on renal function through necrosis and apoptosis of renal cells (8). Therefore, apoptosis reduction in renal tissue is one of the principal mechanisms that is noteworthy in AKI treatment. 

To provide an evidence for the effectiveness of hEnSC transplantation in AKI treatment, the present study was designed based on the hypothesis that administration of these cells can modulate the expression levels of *Bcl-2* and Tnf-α in the renal tissue from cisplatin-induced AKI rats. Such changes in transcriptional activities of *Bcl-2* and Tnf-α are related to apoptosis inhibition, which can result in the improvement of renal pathology and function in rats receiving cell transplants after cisplatin injection.

## Materials and Methods

The experimental study was performed on 48 male Wistar rats (150-200 g). The animals were kept in plastic cages. They had unlimited access to water and food, and were weighed daily. Ethical principles were followed in accordance with Tabriz University of Medical Sciences guidelines (IR.TBZMED.VCR.REC.1397.049). AKI was induced using intraperitoneal (IP) injection of cisplatin (Mylan, France). Animal care was provided by animal house of Kharazmi University at 22.0 ± 2.0˚ C.

A total of 48 rats were randomly divided into four groups (12 each). Group I (intact group) received no treatment; group II (model group) received 5 mg/kg of body weight cisplatin (IP), which was used to induce AKI; group III (PBS group) received 200 µl of phosphate-buffered saline (PBS, Invitrogen, USA) in caudal vein 3 hours after cisplatin injection; and group IV (cell group) received about 1 million hEnSCs suspended in PBS (200 µl) in caudal vein 3 hours after cisplatin injection. The rats were anesthetized by IP injection of 80-100 mg/kg ketamine and 5-10 mg/kg xylazine. The rats were sacrificed on the 3^rd^ and 5^th ^day after cisplatin injection. After anesthesia, blood collection was performed from the heart and the kidneys were collected rapidly for subsequent analyses.

### Isolation of human endometrial stromal/stem cells

The cell donor was an infertile woman who had referred to the hospital seeking treatment. A written informed consent form was obtained from her prior to the procedure. Endometrial biopsies for hEnSC isolation were obtained from the fundal region of the uterine cavity. First, endometrium was scraped from the myometrium and then washed in PBS. Mechanical minced tissue was digested using 1 mg/ml collagenase type I (Gibco, USA) and 25 mM 4-(2 hydroxyethyl)-1 piperazineethanesulfonic acid (HEPES) in Hank’s balanced salt solution (HBSS, Merck, Germany) at 37ºC for 30-45 minutes. Glandular epithelial components were removed by means of cell strainers (70 and 40 μm, Merck, Germany). Cell suspension was centrifuged for cellular plaque deposition and plated in DMEM/F12 medium (Merck, Germany) supplemented with 10% fetal bovine serum (FBS), 1% antibiotic Pen/Strep (Merck, Germany). Medium change was performed every 2-3 days and cellular passage was carried out when cultures reached about 80-90% confluency. The 3^rd^ to 5^th^ passage cells were used for injection.

### Flow cytometric analysis of human endometrial stromal/stem cells

After the 3^rd^ passage, cells were characterized for expression of surface markers by flow cytometry (BD FACS Calibur, USA). Following hEnSCs fixation (2% paraformaldehyde in PBS, 4˚ C, 1 hour), cells were washed and incubated using the following PerCP- or phycoerythrin (PE)- or fluorescein-isothiocyanate (FITC)-conjugated antibodies (4˚ C, 1 hour): CD73-PerCP (5 µl, BD Pharmingen, USA), CD90-FITC (3 µl, Exbio, Czechia), CD105-PE (3 µl, Exbio, Czechia), CD146-PE (3 µl, BD Biosciences, USA), CD31-PE (3 µl, Immunostep, Spain) and CD34-PE (3 µl, Exbio, Czechia). CD31 and CD34 are endothelial and hematopoietic markers, respectively. Negative control was FITC-conjugated mouse IgG1. Data were analyzed using FlowJo™ v10.6.1 software.

### Renal function

On days 3 and 5 after transplantation of cells, rats were anesthetized and blood samples were collected from the rat hearts. After 30 minutes, blood samples were centrifuged (3000 rpm, 10 minutes) and upper layer or serum was isolated. Samples were stored at -20˚C until analysis. To access the renal function, we determined levels of blood urea nitrogen (BUN), serum creatinine (SCr), serum sodium (Na), and potassium (K) on days 3 and 5 after cellular therapy. Biochemical markers of the sera were measured using a Selectra Pro M (ELITechGroup, USA) and a starlyte electrolyte analyzer (Diamond Diagnostics Inc., USA).

### Histopathological assay by hematoxylin and eosin staining

Histological examination of the kidneys was performed on day 5. Kidneys were washed twice with PBS, quickly fixed in 10% formalin solution (Merck, Germany), then processed and embedded in paraffin (Merck, Germany). Finally, 5-µm thick sections were obtained. Histological examination was performed using hematoxylin (Merck, Germany) and eosin (Merck, Germany) staining. The slides were observed under a light microscope (Zeiss, Germany). The presence of pathologic tubular damage (including apoptosis, necrosis, lumen dilation, debris in the lumen and nuclear fragmentation) was scored in 10 non-overlapping fields (100 tubules) for each kidney section by a researcher who was blind to the experimental groups. The score 0 represents no tubular damage, 1= tubular injury in no more than one third of the tubule cells, 2= one third to two thirds of the tubular cell injury, and 3= more than two thirds of the tubular injury. Finally, total pathologic score was obtained by adding all 100 scores. The maximum score was 300.

### Immunohistochemical analysis

TUNEL assay was performed on paraffin-embedded tissues using the terminal deoxynucleotidyl transferase– mediated dUTP nick-end labeling (TUNEL) method (In Situ Cell Death Detection Kit, Roche, Germany) according to the manufacturer’s instruction.

Paraffin sections (5-μm thick) of kidneys were rehydrated in a series of alcohol and water after deparaffinization with xylene. Then sections digested by proteinase K solution (10 minutes, 37˚C) were washed in PBS for 2 minutes, and after blocking (i.e. blocking endogenous peroxidase, 3% H_2 _O_2_ for 30 minutes), were incubated with TdT enzyme solution (60 minutes, 37˚C). A stop/ wash buffer (30 minutes, 37˚C) was used to terminate the reaction. The reaction was visualized using a 3, 3′-diaminobenzidine (DAB) chromogen (Pro Taqs, Cat#300155400) and tissue counterstaining was performed using hematoxylin. Sections were observed for TUNEL+ cells per high-power field (HPF) under a light microscope (Zeiss, Germany). For each kidney, 10 fields were selected randomly at 40x magnification, and the number of TUNEL-positive cells and the total number of cells in the glomeruli and tubules were counted using ImageJ software (LOCI, University of Wisconsin). The percentage of apoptotic cells was evaluated in a blind manner. Data were analyzed using GraphPad software.

### Real-time polymerase chain reaction

The kidney samples were minced and RNA was extracted using a Maxcell extraction kit (Iran). Synthesis of cDNA was performed using First Strand cDNA synthesis kit (Maxcell, Iran). RNA/primer mixture (14 μl) including 1 μl random hexamer, 1 μl of 10 mM dNTP mix, total RNA (volume based on normalization calculations), and DEPC water (up to 14 μl), was incubated for 5 minutes at 65˚C and was then placed on ice immediately for 1 minute. Next, 1 μl Dia star (reverse transcriptase, RT), 1 μl of 8 mM DTT and 4 μl of 5X RT buffer were mixed to prepare reaction master mix. Reaction master mix was added to RNA/ primer mixture and then placed in the thermal cycler as follows: 50˚C, 60 minutes, and 95˚C, 5 minutes. Real time-polymerase chain reaction (PCR) was carried to in duplicate. Reaction volume was 25 μl consisting Real Q Plus 2X Master Mix Green (Ampliqon, Denmark), 2 μl of primer mixture and 2 μl of cDNA under the following thermal cycling: 5 minutes at 50˚C, 5 minutes at 95˚C and 40 cycles of denaturation for 30 seconds and annealing/extension at 60-62˚C. Relative expression levels of *Bcl-2* and *Tnf-α* mRNA were calculated using the 2^-ΔΔCT^ method. *Gapdh* gene was considered as internal control. Primer sequences are showed in Table 1.

**Table 1:** The primer sequences were used in the real-time polymerase chain reaction


No.	Gene symbol	Gene name	Primer sequences (5´-3´)	TM	GC%

1	*Bcl-2*	B-cell lymphoma 2	F: CTTTGAGTTCGGTGGGGTCA	59.35	55.00
			R: TCCACAGAGCGATGTTGTCC	59.35	55.00
2	*Tnf-α*	Tumor necrosis factor alpha	F: GGCAGGTTCTGTCCCTTTCAC	61.78	57.14
			R: TTCTGTGCTCATGGTGTCTTTTCT	59.30	41.67
3	*Gapdh*	Glyceraldehyde 3-phosphate dehydrogenase	F: CAAGTTCAACGGCACAGTCA	57.30	50.00
			R: CCCCATTTGATGTTAGCGGG	59.35	55.00


### Statistical analysis

Data are shown as mean ± SEM. Data analyses were performed using GraphPad Prism 8.0.2 (GraphPad Software Inc., USA). We compared the differences among the groups using One-way analysis of variance (ANOVA) with Tukey test. Analyses of body weight between days 1 and 5 were done in each group using two-way ANOVA. Expression levels of mRNA were analyzed using the comparative Ct method. P<0.05 were considered as statistically significant. 

## Results

### Human endometrial stromal/stem cells culture and characterization

**Fig.1:**
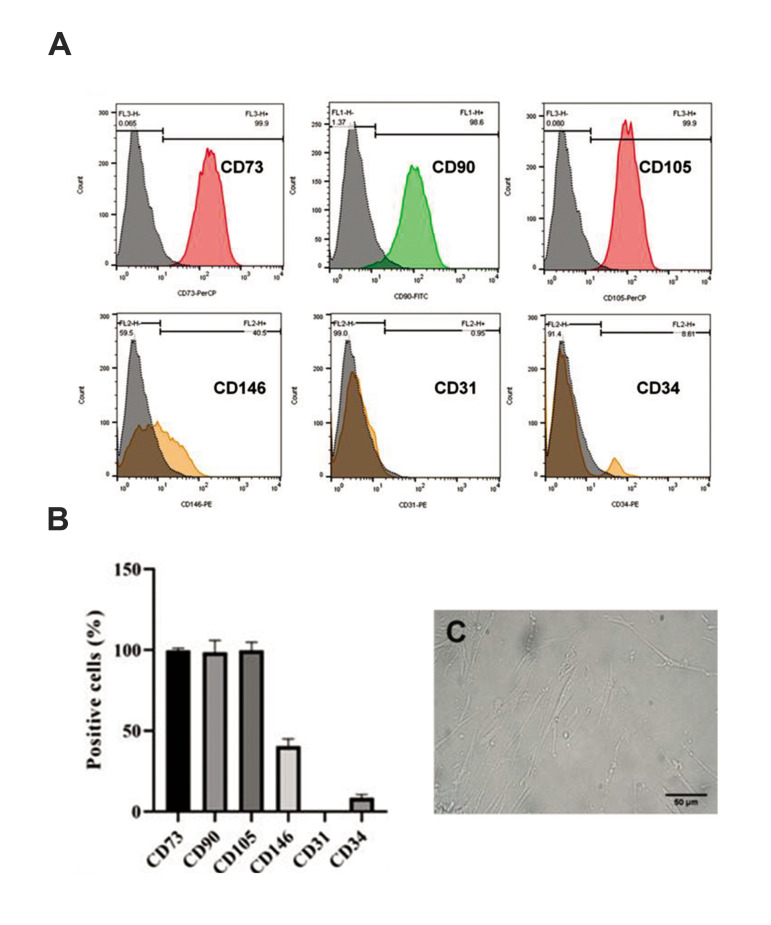
Flow cytometry and morphological feature of extracted hEnSCs after the 3^rd^ passage. **A, B.** HEnSCs are positive for MSC markers and are negative for hematopoietic and endothelial markers. Data show the mean of positive cells (%) ± SEM. **C.** HEnSCs are spindle-shaped and relatively elongated cells (scale bar: 50 µm). hEnSCs; Human endometrial stromal/ stem cells and MSC; Mesenchymal stem cell.

Isolation of undifferentiated hEnSCs was easily performed due to their adherence to plastic flasks. The results of hEnSCs immunophenotyping at passage 3 are presented in histograms in Figures 1A and B. HEnSCs were positive for MSC markers, including CD73, CD90 and CD105, as well as for the marker of endometrial stem cell, CD146. Although they expressed high levels of these markers, they did not express CD31 and CD34, which are endothelial and hematopoietic progenitor cell markers, respectively (Fig .1A, B). HEnSCs were spindle-shaped and were relatively elongated (Fig .1C). These cells were cultured and reached 80-90% confluency before passage.

### Changes of body weight

**Fig.2:**
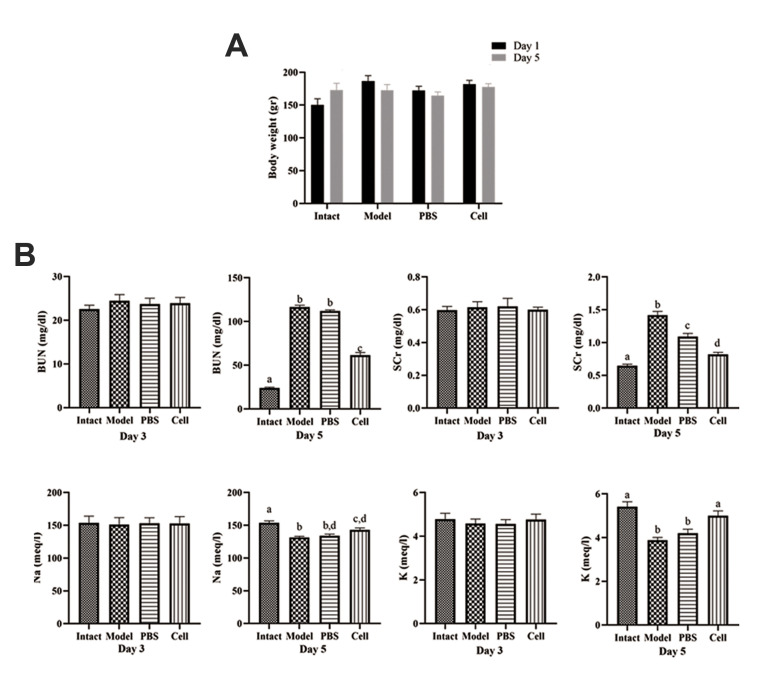
Body weight and serum biochemical changes after hEnSCs injections. All animals except for the intact group received cisplatin (5 mg/ kg, IP) on day 1. After 3 hours, the cell group received 1×10^6^ hEnSCs in 200 µl PBS. **A.** Mean body weights of the treated animals. **B.** The collected serum samples are from days 3 and 5 after cisplatin. Following hEnSCs injections the levels of BUN, SCr, ion Na, and K were analyzed as shown here. Each column presents mean ± SEM. a-d; Show significant differences between groups (P˂0.05), hEnSCs; Human endometrial stromal/stem cells, IP; Intraperitoneal, PBS; Phosphate-buffered saline, BUN; Blood urea nitrogen, SCr; Serum creatinine, Na; Sodium, and K; Potassium.

In the present study, all rats were weighed daily at the appointed time. Body weights were reduced until day 5 in all groups except for the intact group. Body weight for intact group was 150.00 ± 9.55 g and 172.90 ± 10.30 g on days 1 and 5, respectively. Cisplatin administration induced weight loss in model and PBS groups on day 5 as compared to day 1 (172.50 ± 8.99 g, and 186.8 0 ± 8.14 g, vs. 164.50 ± 5.57 g, and 172.00 ± 6.70 g, respectively). Interestingly, a lower body weight loss in cell-transplanted rats (177.50 ± 5.18 g on day 5, 182.00 ± 5.174 g on day 1) as compared to model and PBS groups, may indicate the therapeutic effects of hEnSCs on the disturbance of tubular concentration function (Fig .2A).

### Biochemical analysis of renal function

Successful cisplatin-induced AKI was determined by measuring biochemical markers in blood samples obtained on days 3 and 5. The observed increase in biochemical markers indicated the development of AKI.

The BUN levels in all groups were almost similar on the 3^rd^ day without a statistically significant difference. On day 5, the BUN levels in model and PBS groups were higher (116.60 ± 2.12 and 112.00 ± 1.35 mg/dl, respectively) as compared to those of the hEnSC-transplanted and intact rats (61.53 ± 3.07 and 24.04 ± 0.89 mg/dl, respectively) on day 5. The highest level of BUN was observed in the model group, but cell injection resulted in a significant decrease in BUN levels (Fig .2B).

The changes of SCr quantities were similar to those of BUN levels. SCr increased significantly in model, PBS, and hEnSC-transplanted groups as compared with the healthy intact animals (0.65 ± 0.02 mg/dl) on day 5. Also, a significant decrease (P<0.05) was observed in the SCr level in the group receiving hEnSCs compared to the model and PBS groups (0.82 ± 0.03 vs. 1.42 ± 0.06, 1.09 ± 0.05 mg/dl respectively) on day 5 (Fig .2B).

Serum Na and K levels on day 3 were similar among the groups (about 150 milliequivalent (meq)/l and 4.5 meq/l, respectively). On day 5, there were significant decreases (P<0.05) in the mean values of Na levels in the model, PBS and the cell groups (131.60 ± 1.61, 134.30 ± 2.37 and 143.00 ± 3.13 meq/l, respectively) as compared to that of the intact group (153.80 ± 2.92 meq/l), but the difference between PBS and the cell groups was not significant. Also, cell treatment resulted in the mild increase of Na level (Fig .2B). Moreover, on day 5, we observed a significant decrease (P<0.05) in the mean K levels in the model and PBS (3.87 ± 0.13 and 4.20 ± 0.19 meq/l, respectively) as compared to those of the intact and the cell groups (5.41 ± 0.23 and 5.00 ± 0.22 meq/l, respectively). In addition, the quantities of K in rats receiving hEnSCs were not significantly different compared with those of the intact group on day 5 (Fig .2B).

### Pathologic changes

**Fig.3:**
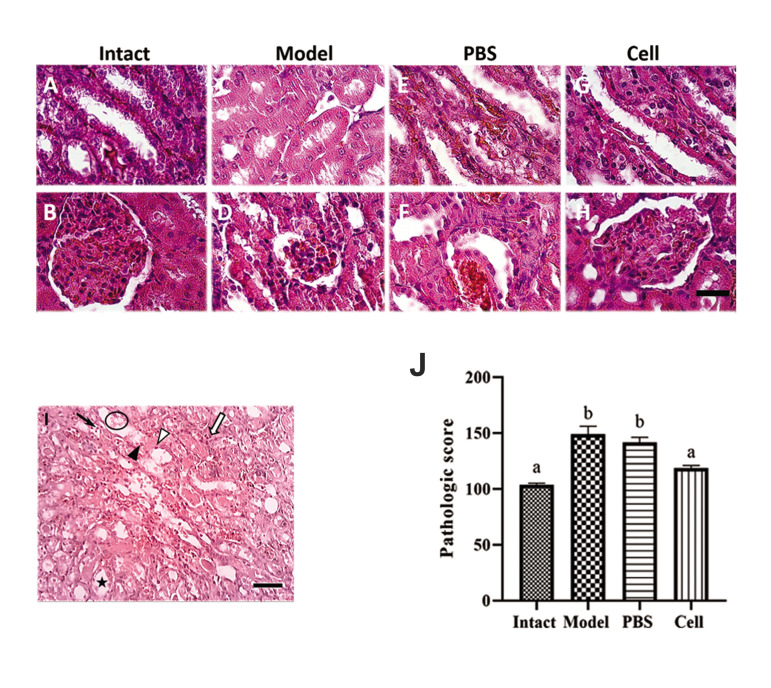
Pathologic analyses of kidneys after hEnSCs injection on day 5. **A-H.** HEnSCs injection led to significant reduction of pathologic injuries in renal tissue (original magnification: ×40, scale bar: 50 µm). **I.** Photomicrograph shows tubular injuries: cell debris in the lumen (circle), lumen dilation (star), regenerative change in tubular cells or flattening of the epithelium lining the tubules (white triangle), nuclear fragmentation (white arrow), apoptosis (black arrow) and necrosis (black triangle) (original magnification: ×10, scale bar: 100 µm). **J.** Histogram shows pathologic scores in different groups. a, b; Show significant differences between groups (P˂0.05), hEnSCs; Human endometrial stromal/stem cells, and PBS; Phosphate-buffered saline.

Examination of renal sections was performed in the cortex and medulla regions on day 5 (Fig .3, original magnification:×40). Light microscopic analyses showed significant changes in the renal tissue on day 5 after cisplatin injection. Histological examination of the kidney tissues obtained from both model and PBS groups showed proximal and distal tubular injury with cell debris in the lumen, lumen dilation, regenerative changes in tubular cells, nuclear fragmentation, and apoptosis and necrosis of the epithelial cells (Fig .3C-F). On day 5 following hEnSC injection, a significant improvement was observed in renal glomeruli and tubules, thus the pathologic scores for tubular damage were 118.8 ± 2.15 for the cell group versus 149.1 ± 7.02 and 141.5 ± 4.68 for the model and PBS groups, respectively. Similar to the beneficial effects on the serum biomarkers associated with renal function, hEnSCs had a significant effect on renal pathology, as they improved the renal pathologic scores (Fig .3).

### Evaluation of apoptosis in tubular epithelial cells and glomeruli after cell therapy

**Fig.4:**
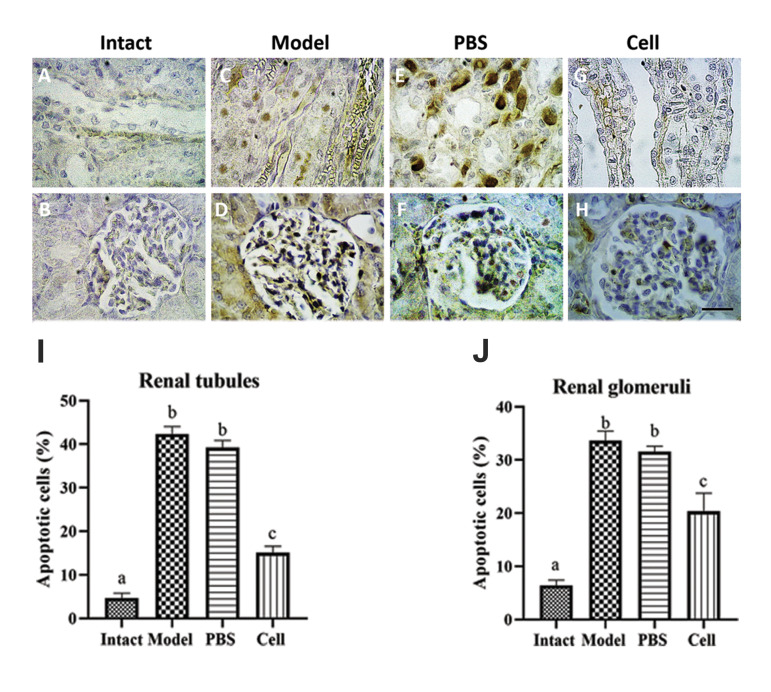
Results of TUNEL test after hEnSCs injection on day 5. **A, B.** Intact group, **C, D.** Model animals receiving the cisplatin, **E, F. **PBS group receiving PBS 3 hours after cisplatin injection, **G, H. **Cell group receiving hEnSCs 3 hours after cisplatin injection. TUNEL (+) cells were observed in tubules and glomeruli (original magnification: ×40, scale bar: 50 µm). **I, J. **Histograms showed the percentage of TUNEL (+) cells. Each column presents mean ± SEM. a-c; Show significant differences between groups (P˂0.05), hEnSCs; Human endometrial stromal/stem cells, and PBS; Phosphate-buffered saline.

Apoptosis in paraffin embedded tissues was detected using TUNEL kit and data were analyzed via ImageJ Software. On day 5, the percentage of TUNEL-positive apoptotic cells in tubules and glomeruli increased significantly in both model and PBS groups in comparison to the intact group (Fig .4A-F) (33.67 ± 1.79, 31.53 ± 1.05 vs. 6.37 ± 1.05 in glomeruli and 42.33 ± 1.72, 39.23 ± 1.61 vs. 4.67 ± 1.17 in tubules). HEnSC-transplantation resulted in a marked reduction of apoptotic cells in the renal tissue (Fig.4G, H), suggesting an inhibitory effect of the hEnSCs on cisplatin-induced apoptosis. The results of the percentage of apoptotic cells in different groups are shown in histograms Figure 4 I and J.

### Effects of treatment with hEnSCs on renal mRNA expression of *Bcl-2* and *Tnf-α*

**Fig.5:**
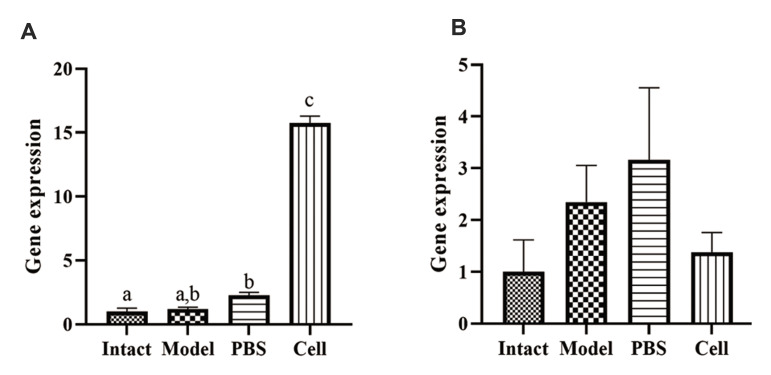
Changes of gene expression in renal tissue after hEnSC transplantation on day 5. **A. ***Bcl-2* and **B. ***Tnf-α* expression in intact, model, PBS and cell groups on day 5. Values are presented as mean ± SEM. a-c; Show significant differences between groups (P˂0.05), hEnSC; Human endometrial stromal/stem cells, and PBS; Phosphate-buffered saline.

Investigation of direct effect of hEnSC treatment on renal production of *Bcl-2* and *Tnf-α* was done using real-time PCR. The expression levels of the mRNA for *Bcl-2* (anti-apoptotic and anti-inflammatory gene, intrinsic apoptotic pathway) and *Tnf-α* (pro-apoptotic and pro-inflammatory cytokine gene, extrinsic apoptotic pathway) changed after cisplatin and hEnSC injection in comparison to those of the intact group on day 5 (Fig .5). The mean *Bcl-2* levels in the intact, model, PBS and cell groups were 1.00 ± 0.28, 1.21 ± 0.12, 2.28 ± 0.22, and 15.74 ± 0.55, respectively. Model, PBS, and the cell groups showed a meaningful increase (P<0.05), when compared to the intact group, but the difference between the model and PBS groups was not significant. The mean *Tnf-α* levels were 1.00 ± 0.61, 2.35 ± 0.71, 3.16 ± 1.39, and 1.38 ± 0.39 in the intact, model, PBS, and cell groups respectively. Cisplatin treatment caused an increase in *Tnf-α* levels but hEnSCs transplantation lead to a decrease in *Tnf-α* level in the cell group. The difference between the experimental groups in terms of *Tnf-α* level was not significant.

## Discussion

The majority of stem cell therapy studies have focused on the use of bone marrow (BM)-MSCs. The useful effects of these cells on the function of kidneys have clearly been demonstrated in different AKI animal models by various groups (24). 

To date, BM has been considered as the most ideal source of stem cells for transplantation in regenerative medicine; however, the disadvantages related to BM-MSC such as the invasive method of extraction, age-related decline of stemness and proliferative ability, the required anesthesia for the donors, etc. restrict its applications. Human endometrium also is considered as a source for stem cells. In 2010, about 430,000 inpatient hysterectomies were carried out in the US alone, demonstrating that it is a significant source of endometrial stem cells. Fortunately, hEnSCs exist in the superficial layers of this tissue, so these cells are accessible without impairment of endometrial function (25-27). In regenerative medicine, BM-MSCs are more suitable for hard tissue engineering, such as bone, whereas EnSCs are predicted to be more suitable candidates for soft tissue engineering including kidney (22). 

Obtaining endometrial specimen does not require anesthesia or sedatives and causes minimal morbidity and pain. One of the clinical limitations of using MSCs is age-related proliferative ability, whereas, the proliferative capacity of hEnSCs is not impaired in the elderly (28). The effects of endometrial MSC-like or hEnSCs on renal function and apoptosis reduction in AKI has not been studied thoroughly so far. To investigate such effects, we injected hEnSCs after induction of AKI using a single dose of cisplatin. Subsequently, we determined the levels of serum biomarkers, renal pathology, and apoptotic cell percentage in renal tubules and glomeruli, as well as expression of *Bcl-2* and* Tnf-α* genes using real-time PCR.

The results of biochemical and histological analyses showed that 5 mg/kg body weight cisplatin successfully induces AKI, while hEnSCs infusion leads to the recovery of the AKI-associated signs. Following cell therapy, the levels of serum biomarkers were closer to those of the normal state and the pathologic score was reduced as well. Several reports in cisplatin-induced AKI model have shown that MSCs ameliorated the cisplatin effects on renal tissue (7, 19, 29), nonetheless, beneficial effects of MSCs on the recovery of cisplatin effects are still controversial (30). 

It has previously been shown that cisplatin (5 mg/ kg) results in the increase of urea, creatinine, K level, and significant changes in monkey renal tissue on day 4. These results demonstrated that intra-renal arterial injection of autologous BM-MSCs ameliorated the levels of urea and creatinine. BM-MSCs were not observed to have a significant influence on pathologic scores of kidneys, hyaline casts and fibrosis scores on days 4 and 28 after cisplatin injection (30). Similarly, we have shown that BUN, SCr and Na underwent changes, but the K level and the renal histologic scores improved after cell transplantation. 

Sun et al. investigated therapeutic effects of human urine-derived stem cells (USCs) in cisplatin-induced AKI in a rat model. They induced AKI using IP injection of 5 mg/kg cisplatin and at 24 hours later injected 2×10^6^ USCs/ 0.2 ml PBS via tail vein. One of the criteria to evaluate in their study was histological changes. Tubular damage score and the number of injured glomeruli decreased markedly after USC injection on day 4 (31). Our histopathologic results were similar to those of their study, because hEnSCs improved histological scores in our rat model of cisplatin-induced AKI significantly.

Ultimate damaging impact of cisplatin on the kidneys, results from apoptosis and necrosis of renal cells (8). Different stimuli including intracellular (reactive oxygen species [ROS]-induced mitochondrial damage) and extracellular (activation of death receptors) lead to cell death (32). Cisplatin induces renal cell apoptosis via p53-mediated activations of caspase-2, 3, 8, while TNF-α synthesis by phosphorylation of p38 MAPK may be the basis of necrosis in tubular cells (33). Also, it has been shown that outer mitochondrial membrane damage and activation of apoptotic intrinsic pathway can be induced by cisplatin. Bcl-2 family plays a significant role in nephrotoxicity of cisplatin. Treatment by cisplatin results in the reduction of Bcl-2 and BAX ratio, upregulation of apoptotic genes, degradation or decrease of anti-apoptotic proteins, and increase of pro-apoptotic proteins and inflammatory mediators, such as TNF-α (34-38). Therefore, reduction of renal cell apoptosis is one of the important mechanisms to be considered in the treatment of AKI.

Human umbilical cord-derived MSCs (HUC-MSCs) reduce cell apoptosis and help repair tubular epithelial cells via upregulation of *Bcl-2* and *Bmp-7* (39). MSCs attenuate cisplatin-induced nephrotoxicity by modulating renal inflammation. MSCs significantly recover cisplatin-induced renal failure by apoptosis suppression in p53-dependent and paracrine manner (29, 40). These results, as reported in previous studies, support our findings. The present study demonstrated that the transplantation of hEnSCs result in upregulation of *Bcl-2* and downregulation of *Tnf-α*, which is consistent with apoptosis decrease and improvement of renal function. These findings suggest a relationship between the change in the intrinsic and extrinsic pathways of apoptosis and the infusion of hEnSCs. Administration of hEnSCs leads to a significant decrease of TUNEL-positive cells after the cisplatin injection. Our findings suggest that one of the important renoprotective effects of hEnSCs may depend on the inhibition or reduction of apoptosis. 

## Conclusion

Endometrium is a potential source of stromal/stem cells. These cells can be extracted without ethical and technical problems. In the present study, transplantation of hEnSCs changed the expression of *Bcl-2* and *Tnf-α *in renal tissue of animals with cisplatin-induced AKI. According to the findings of biochemical assays, renal pathology evaluation, and gene expression analyses, hEnSCs may be involved in apoptosis inhibition in kidneys and therefore in the improvement of their function in this model of AKI. Further research on changes of other biomarkers, such as cystatin c and NGAL, other pathological and functional aspects of the kidney, and also details of apoptotic pathways are necessary to confidently consider the clinical application of hEnSCs for the treatment of renal diseases.
